# From natural theology to the extended synthesis: Historical milestones and conceptual expansions in evolutionary biology

**DOI:** 10.1590/1678-4685-GMB-2025-0179

**Published:** 2026-02-13

**Authors:** Francisco Prosdocimi, Marco Garbin, Francesco Dondero

**Affiliations:** 1 Universidade Federal do Rio de Janeiro, Instituto de Bioquímica Médica Leopoldo de Meis, Laboratório de Biologia Teórica e de Sistemas, Rio de Janeiro, RJ, Brazil.; 2 Università del Piemonte Orientale Vercelli Novara Alessandria, Department of Sustainable Development and Ecological Transition, Vercelli, Italy.; 3 Università del Piemonte Orientale Vercelli Novara Alessandria, Department of Science and Technological Innovation, Alessandria, Italy.

**Keywords:** Evolutionary biology, history of science, Darwinism, extended evolutionary synthesis, philosophy of science

## Abstract

This article explores the historical development of evolutionary biology-from Natural Theology to the Modern Synthesis (MS)-and the ongoing debate around the Extended Evolutionary Synthesis (EES). Over the past 2,500 years, evolutionary thinking has emerged from the interplay between empirical discoveries and dominant philosophical paradigms. Beginning with Aristotle and Saint Augustine, we trace how Darwin and Wallace introduced a scientific framework grounded in natural mechanisms. In the early 20th century, the MS unified Mendelian genetics and Darwinian selection, forming a gene-centered model of evolution focused on mutations and population dynamics. In recent decades, discoveries in epigenetics, phenotypic plasticity, symbiosis, niche construction, and cultural inheritance have challenged the explanatory scope of MS. The EES seeks to incorporate these processes not by discarding Darwinian principles, but by reinterpreting them through a systems biology lens. This mostly represents a conceptual shift in focus: from linear, gene-driven causality to multilevel, reciprocal, and environmentally embedded dynamics. While gaining traction, the EES has been criticized for its lack of formal models and predictive frameworks, remaining a contested proposal. Ultimately, evolutionary biology continues to evolve as a powerful scientific tradition, driven by humanity’s enduring quest to understand the origins and evolution of life on Earth.

## Introduction: Evolutionary theory as the cornerstone of contemporary biology

The comprehensive corpus of evolutionary thinking remains the central theoretical foundation of biology, acting as the unifying principle that bridges diverse subfields of the life sciences. Since the publication of *On the Origin of Species* by Charles Darwin, in 1859, evolutionary theory has provided the essential mechanistic framework for understanding the complexity, diversity, and adaptive features of living systems (Mayr, 1982; Darwin, 1859; Browne, 1995). Whether addressing molecular interactions or ecological networks, evolutionary concepts continue to inform how scientists analyze biological forms and functions.

Today, evolutionary theory is not merely a historical narrative but a predictive and integrative system. It is indispensable for explaining patterns of genetic inheritance, the biogeographical distribution of organisms, developmental pathways, disease susceptibility, and even the evolution of resistance to antibiotics and antivirals (Nesse et al., 2010; Futuyma and Kirkpatrick, 2017). Core mechanisms-mutation, gene flow, genetic drift, and natural selection-together account for both the shared ancestry of life and the ongoing diversification of species (Dobzhansky, 1973). These principles serve not only as explanatory tools but also as a basis for applied advances in medicine, agriculture, conservation, and biotechnology (Laland et al., 2015).

What distinguishes evolutionary theory is its remarkable scope: it connects genes to ecosystems, and individuals to populations, while retaining a consistent framework for interpreting both micro- and macroevolutionary change. Yet, evolutionary biology is far from static. It continues to evolve in response to new empirical findings and conceptual challenges. Increasingly, researchers acknowledge that phenomena such as developmental plasticity, epigenetic regulation, niche construction, and symbiotic interactions are not peripheral but central to evolutionary change (West-Eberhard, 2003; Jablonka and Lamb, 2005; Gilbert et al., 2012a).

A productive avenue for addressing these developments lies in the intersection of evolutionary biology with the history and philosophy of science. As Thomas Kuhn (1962) suggested, sometimes scientific progress can be shaped by paradigm shifts rather than mere accumulation of data. In evolutionary biology, we can find those shifts (i) in the displacement of natural theology by natural selection, (ii) in the reconciliation of Darwin-Wallace theory with Mendelian genetics during the Modern Synthesis, and more recently, (iii) in the rise of Evo-Devo, systems biology, and the still ongoing effort to construct an Extended Evolutionary Synthesis (Pigliucci and Müller, 2010; Love, 2015). We acknowledge that some historians of science, such as Bowler (1988, 2009a, b), Kohn (1985), and Desmond and Moore (1991, 1994), have emphasized the gradual character of this transition, noting that some evolutionary ideas predated 1859 and that natural selection itself gained widespread acceptance only decades later. Nonetheless, together with Mayr, Ruse and Gould, we maintain that Darwin and Wallace’s proposal of common ancestry and his “tree of life” framework represented a genuine paradigmatic transformation: it reoriented biology away from static and typological conceptions and toward a historical and genealogical understanding of life’s diversity. These transformations reflect not only scientific advancements but also shifts in underlying metaphysical assumptions-such as moving from typological to population thinking (Hull, 1974), or from gene-centric models to more organismal and ecological perspectives (Noble, 2013; Laland et al., 2015).

In the post-genomic era, evolutionary biology faces new challenges and opportunities. The traditional modern synthesis view of adaptation as dependent solely on changes in allele frequencies have been abandoned. Research in epigenetics has demonstrated that heritable phenotypic variation can arise through chemical modifications to DNA and histones, without altering nucleotide sequences (Jaenisch and Bird, 2003; Bossdorf et al., 2008; Allis and Jenuwein, 2016), therefore maintaining alleles unchanged. Moreover, the concept of phenotypic plasticity reveals that organisms can develop divergent traits from a single genotype in response to environmental cues-sometimes with transgenerational effects (West-Eberhard, 2003; Bonduriansky and Day, 2009). The complexity of gene-to-phenotype mapping has also become evident, as studies in regulatory networks, transcriptomics, and chromatin dynamics have shown that genetic effects are highly context-dependent (Carroll, 2005; Noble, 2013).

Equally transformative is the growing understanding of the microbiome and host-microbe interactions, which has led to the reconceptualization of organisms as *holobionts*-integrated assemblages of host and symbiotic organisms whose collective genomes (the *hologenome*) co-evolve and influence phenotype and fitness (Rosenberg and Zilber-Rosenberg, 2008). These findings challenge the classical boundaries of individuality and inheritance, suggesting that evolutionary models must account for multilevel and collective processes of variation and selection.

Despite these developments, the Extended Evolutionary Synthesis (EES) has yet to crystallize into a fully integrated theoretical model. While its advocates argue for a broader view of evolution that incorporates developmental bias, epigenetic inheritance, plasticity, niche construction, and non-genetic forms of inheritance (Laland et al., 2015), critics contend that the Modern Synthesis is sufficiently flexible to accommodate these insights without fundamental revision (Wray et al., 2014). As a result, the debate continues over whether the EES represents a true paradigm shift or an expansion of existing frameworks.

Evaluating these issues and appreciating the current frontiers of evolutionary thought is essential to retrace the major conceptual milestones that have shaped the history of life in Earth. By revisiting the historical trajectory of ideas-from natural theology to Darwin, from Mendelian genetics to systems biology-we will be in a better point to understand the foundations of contemporary biology and anticipate future directions for evolutionary theory.

## The history and legacy of evolutionary theory

### From natural theology to scientific naturalism

The origins of evolutionary thought in Europe can be traced, as in many areas of philosophy and science, to ancient Greece. Some pre-Socratic philosophers-such as Anaximander, as early as the 6th century BCE-had already proposed initial hypotheses regarding the origin of life and its progression from fish to humans (Bardell, 1994). However, it was Aristotle’s perspective that came to dominate Western thought for the first millennium. Aristotle viewed nature as fundamentally static and immutable, asserting that living species had always existed in their current forms. He posited that all living beings were organized into a hierarchical structure based on increasing complexity, from the simplest organisms to the most complex-a vision that became known as the “Great Chain of Being.”

This hierarchical and teleological view of nature was later absorbed into Christian intellectual traditions in the Western culture through the writings of early Church Fathers, notably Augustine of Hippo. Saint Augustine argued that reason could and should be applied to theological reflection, and in *De Genesi ad Litteram* (*The Literal Meaning of Genesis*, 1982), he maintained that studying the natural world was a way to contemplate the divine mind. For him, “the book of nature” was written by the hand of God and should be read alongside Scripture (Augustine, c. 401 CE). This synthesis of classical philosophy and Christian theology laid the foundations for Natural Theology-a worldview in which empirical investigation of natural phenomena could reveal truths about the Creator. Throughout the Middle Ages, this outlook inspired clerics to engage directly in the study of nature and life. Monasteries and cathedral schools became centers of learning, where advances in natural history, anatomy, and botany were made by priests, physicians, and alchemists. Their work combined careful empirical observation with a mystical conviction that God and Nature were inseparably linked, making the description of plants, animals, and minerals not merely a scientific task but also a devotional act-a way of “reading” God’s creation.

Thus, before the advent of Darwinian evolution, Western conceptions of the natural world were deeply shaped by the synthesis of Aristotelian fixism and Augustinian theology. This tradition, crystallized in the framework of Natural Theology (Paley, 1802; Brooke, 1991), viewed nature as the deliberate and immutable creation of God-an idea reinforced by scriptural interpretation and the symbolic imagery of divine craftsmanship (Lovejoy, 1936). Rooted in institutions such as medieval monasteries and universities, it treated empirical observation as a means of confirming the perfection of creation, rather than questioning it. Yet, by the Renaissance and Scientific Revolution (15th-17th centuries), new empirical findings-such as fossil evidence, the geographic distribution of species, and the existence of hybrids-began to expose tensions within the fixist model (Bowler, 2009a). At the same time, the Copernican heliocentric model disrupted the geocentric, anthropocentric worldview central to Natural Theology, challenging humanity’s privileged place in the cosmos. Enlightenment thinkers further eroded theological authority: Hume (1779) argued that order in nature did not necessarily imply a designer, while Kant (1781) maintained that God’s existence could not be established through empirical means. Likewise, Antonio Vallisneri (1661-1730) advocated a shift from scholastic speculation to empirical observation in natural history, seeking mechanistic explanations for biological phenomena and anticipating the methodological naturalism that would later underpin Darwinian thought (Vallisneri, 1713; Shea, 1974).

However, despite mounting critiques, no unified alternative to Natural Theology had yet emerged. As Thomas Kuhn (1962) later observed, a scientific revolution requires not only the accumulation of anomalies but also the presence of a viable competing framework. In the early 19th century, the fixist view was increasingly undermined by new geological and biological insights, yet it remained without a comprehensive replacement. 

Advances in geology were particularly influential: James Hutton’s Theory of the Earth (1795) prefigured a processual understanding that the geological change happened on Earth over vast timescales, implying an age far older than traditionally conceived. But it was Charles Lyell, that, in his Principles of Geology (1830-1833), explicitly formalized uniformitarianism as a methodological principle, emphasizing that present-day processes are the key to interpreting Earth’s past. Giovanni Arduino (1714-1795), often regarded as the father of stratigraphy, proposed the first systematic classification of the Earth’s crust, laying the geological foundation for later evolutionary theories that required deep time and stratified biological succession (Arduino, 1760; Vai, 2009).

Georges Cuvier’s catastrophism suggested that fossils belonged to species rendered extinct by dramatic events, further destabilizing fixist assumptions, though without endorsing evolutionary change. 

Antonio Vallisneri (1661-1730) championed empirical observation and mechanistic explanations for natural phenomena (Vallisneri, 1713; Shea, 1974), while Lazzaro Spallanzani (1729-1799) experimentally refuted spontaneous generation, laying the groundwork for modern concepts of reproduction and biogenesis (Spallanzani, 1769; Farley, 1972). 

Linnaeus’ *Systema Naturae* (1735) laid the groundwork for biological classification by establishing a binomial nomenclature and hierarchical system that organized living forms into nested categories (Linnaeus, 1735-1758; Larson, 2004). At the same time, Linnaeus, while steadfast in his creationist beliefs, classified humans among primates and acknowledged hybrid forms (so-called *hybrida plantae*) in his *Species Plantarum* (1753) and *Philosophia Botanica* (1751), though he interpreted them within a framework of limited fixity. 

Likewise, Buffon’s Histoire Naturelle (1749-1788) introduced a naturalistic view of species variation and environmental influence, anticipating later discussions on adaptation and heredity (Buffon, 1749; Sloan, 1976). As director of the *Jardin du Roi*, Buffon was Lamarck’s early supervisor, yet he did not support Lamarck’s transformist ideas, often dismissing them as speculative and incompatible with his own concept of limited species ‘degeneration’ (Farber, 1972; Corsi, 1988). 

These intellectual pan-European dialogues on nature’s continuity and transformation shifts set the stage for Charles Darwin and Alfred Wallace, whose theories at last offered a coherent, empirically grounded explanation for the origin, transformation, and diversity of species.

### Darwin and the evolutionary paradigm shift

Defining Darwinism in a singular and comprehensive way is inherently problematic, as noted by Greene (1959), given the conceptual evolution across Darwin’s and Wallace’s writings and the varying rates of acceptance of their independent propositions. The purpose of this section is not to offer a definitive definition, but rather to present Darwinism as understood by the authors-emphasizing those aspects deemed most central to the development of evolutionary thought.

Darwin’s voyage aboard the HMS *Beagle* (1831-1836) provided the foundational empirical data for the development of a compelling and new theory of biological evolution. His detailed observations in South America and the Galápagos Islands revealed significant biogeographical patterns and morphological variation among closely related species (Darwin, 1839; Browne, 1995). These insights, coupled with the influence of Thomas Malthus’s An Essay on the Principle of Population (1798), led Darwin to formulate the principle of natural selection-whereby differential survival and reproduction act upon heritable variation, gradually shaping populations over time (Darwin, 1859; Mayr, 1982).

However, despite returning from the Beagle voyage in 1836 with a wealth of observations, Darwin took more than two decades to publish his theory. During this period, he devoted himself to an expansive, unpublished manuscript-what he referred to as his “big book on species”-and struggled with both the complexity of the evidence and his own doubts about how his ideas would be received. His hesitation was compounded by a desire for exhaustive documentation, resulting in years of additional research on geology, barnacles, and artificial selection before making his evolutionary arguments public.

In 1858, Alfred Russel Wallace independently arrived at similar conclusions, prompting a joint presentation to the Linnean Society (Darwin and Wallace, 1858). This collaborative moment catalyzed Darwin’s decision to publish *On the Origin of Species* in 1859. By then, Darwin had already earned scientific prestige through his geological writings and monographs on barnacles (*Cirripedia*), which displayed his methodological rigor and capacity for meticulous morphological comparison (Darwin, 1851-1854; Desmond and Moore, 1991).

According to Ernst Mayr (2004), Darwin’s contribution can be understood as a synthesis of five core interrelated theories: (1) evolution *per se* (the idea that species change over time), (2) common descent, (3) gradualism, (4) population thinking (emphasis on variation rather than types), and (5) natural selection. Of these, the concept of common descent gained the quickest scientific acceptance due to its power in explaining observed patterns in comparative anatomy, embryology, and biogeography (Mayr, 2004; Ruse, 1979). Under this framework, the Linnaean hierarchy was reinterpreted as reflecting evolutionary relationships rather than static divine archetypes. Homologous structures-such as vertebrate limb bones or embryonic gill arches-gained evolutionary meaning as inherited features from shared ancestors (Amundson, 2005). Table 1 present the major differences that base the paradigm shift between Natural Theology and Darwinian approaches.

**Table 1 -:** Comparative framework of Natural Theology and Darwinism.

Aspect	Natural Theology	Darwinism
View of Nature	Static and immutable creation, reflecting divine perfection	Dynamic and evolving, shaped by natural processes over time
Source of Order	Purposeful design by a Creator	Emergent order from variation, heredity, and natural selection
Role of Empirical Observation	To confirm the perfection of creation and reveal divine craftsmanship	To test hypotheses, explain natural phenomena, and uncover causal mechanisms
Explanation for Diversity	Diversity is part of God’s deliberate design, each species fixed since creation	Diversity arises from modification through descent, adaptation, and speciation
Historical Roots	Aristotelian fixism integrated with Augustinian theology, medieval scholarship, and Paley’s watchmaker analogy	Based on geological, biogeographical, and morphological evidence gathered in the 19th century
View of Humanity	Humanity as a central and privileged creation	Humanity as one species among many, sharing common ancestry with other organisms
Philosophical Orientation	Teleological and theological	Naturalistic and mechanistic

However, Darwin’s theory faced a critical limitation: the absence of a satisfactory mechanism of heredity. Although the Agostinian friar Gregor Mendel had already formulated his laws of inheritance in the 1860s, they remained largely unknown until the early 20th century. Darwin, unaware of Mendel’s work, attempted to fill this gap through speculative models such as pangenesis-the idea that all parts of the body emit tiny particles (gemmules) influencing the germ cells. He also considered the inheritance of acquired traits, echoing Jean-Baptiste Lamarck’s earlier ideas on use and disuse (Darwin, 1871, 1872). In *The Expression of the Emotions in Man and Animals* (1872), Darwin argued that emotional expressions, once useful, could become habitual and even heritable, implying a soft form of acquired inheritance.

This aspect of Darwin’s thought reveals a historical irony: while often framed as an antagonist to Lamarck, Darwin was one of the most influential transmitters of Lamarckian ideas in the Victorian scientific community. Scholars such as Burkhardt (1995), Bowler (2009b), and Gissis (2009) have shown that Darwin’s reliance on use and disuse mechanisms was not merely a residual idea but a strategic solution to the problem of variation and adaptation in the absence of a known genetic mechanism. Darwinian evolution, as first conceived, did not displace Lamarckian inheritance but rather incorporated it as a provisional explanatory tool (Ellegård, 1958).

It is also important to recognize that Lamarckian ideas were never hegemonic within the scientific community, even in the early 19th century. And that is why it has not been considered here as a paradigm shift. While Lamarck’s theory of *transformisme* was innovative in positing that organisms change gradually in response to their environment, it was met with skepticism from contemporaries such as Georges Cuvier, who advocated for the fixity of species and catastrophism (Cuvier, 1812; Coleman, 1964). The inheritance of acquired characteristics lacked empirical support and was philosophically inconsistent with emerging trends in mechanistic biology. Thus, Lamarck’s influence, although historically significant, was limited and uneven at his time (Mayr, 1982).

Darwin’s own use of such mechanisms was less a wholesale endorsement of Lamarck than a pragmatic attempt to address the gaps left by the absence of a mechanistic model of heredity. His openness to diverse explanatory possibilities reflects the empirical and exploratory spirit of his science, but it also highlights the incomplete nature of early evolutionary theory. While Darwin succeeded in explaining the branching pattern of life (tree of life) and the basic mechanisms for natural selection, he was unable to convincingly explain the origin and maintenance of variation-what he termed the “laws of variation”-a lacuna that would only be addressed decades later with the rediscovery of Mendel’s laws and the rise of population genetics.

In this regard, Darwin’s theory can be seen as both revolutionary and transitional. It transformed biology into a historical science grounded in natural causes, yet it relied on pre-genetic assumptions about heredity. 

### Mendel, the Neo-Mendelians, and the foundations of genetics

At the turn of the 20th century, the rediscovery of Gregor Mendel’s foundational work on inheritance catalyzed a transformative moment in biology. Independently and almost simultaneously, three European botanists-Hugo de Vries in the Netherlands, Carl Correns in Germany, and Erich von Tschermak in Austria-replicated Mendel’s experiments and acknowledged the statistical regularities of trait inheritance first published in 1866 in *Versuche* über *Pflanzen-Hybriden* (Mendel, 1866). While de Vries (1901) initially failed to credit Mendel, Correns later emphasized that it was Mendel who had laid down the principles of segregation and dominance. Tschermak, although more controversial in terms of priority, contributed to the empirical validation of Mendel’s patterns through systematic crossbreeding (Olby, 1985).

De Vries’s interpretation of inheritance, however, did not align entirely with Darwin’s evolutionary framework. In his *Mutationstheorie*, he proposed that new species arise from sudden, discontinuous mutations, particularly based on his observations of *Oenothera lamarckiana* (evening primrose), a plant with chromosomal irregularities that appeared to support this model (de Vries, 1901-1903). This theory of saltational evolution-evolution by jumps-stood in contrast to Darwin’s insistence on gradualism and continuous variation, becoming a key source of friction between early Mendelians and Darwinists (Bowler, 1983; Larson, 2004).

The early Mendelians, especially in the UK under the leadership of William Bateson, saw Mendelism as a corrective to what they perceived as the vagueness of Darwinian gradualism. Bateson became Mendel’s most vocal advocate in the English-speaking world, translating his papers, coining the term “genetics,” and introducing terms such as “allele,” “homozygote,” and “heterozygote” (Bateson, 1909). He championed the idea that variation was not smooth and continuous, as Darwin assumed, but rather discontinuous, driven by identifiable and heritable units. For Bateson and his followers, Darwin’s reliance on blended inheritance and unobservable small variations seemed outdated and inadequate (Bateson and Saunders, 1902).

The Mendelian view also found support among biometricians such as Charles Davenport in the United States, although their focus on statistical correlations between traits began to diverge from the mechanistic, gene-centered view advanced by Bateson (Provine, 1971). Meanwhile, many Darwinian naturalists remained skeptical of Mendelism. They viewed the Mendelian model-based on a limited set of traits in peas and other experimental plants-as too simplistic to explain the complexity of natural populations and macroevolutionary patterns.

This division crystallized into a wider intellectual conflict that some historians have referred to as a “war of the biometricians and the Mendelians” (Porter, 1977). On one side, biometricians such as Karl Pearson and W. F. R. Weldon, working in the Galtonian tradition, applied statistical methods to large population datasets, emphasizing continuous variation, the role of environmental influences, and the heritability of complex traits. On the other side, Mendelians such as Bateson highlighted discrete hereditary units (genes) and the role of mutations, initially rejecting gradualist accounts of evolutionary change (Cock and Forsdyke, 2008; Radick, 2016). Weldon, in particular, was deeply critical of Mendelian application to evolutionary biology, arguing that Mendelian traits rarely mapped onto the kinds of adaptive features shaped by natural selection in wild populations (Weldon, 1902). As Provine (1971) and others have emphasized, these disputes illustrate that the eventual reconciliation of Darwinism and Mendelism into what became the Modern Synthesis was not immediate but rather the product of sustained negotiation and conceptual compromise, rather than linear consensus.

The split between Mendelians and Darwinists extended into broader philosophical questions about the nature of variation, inheritance, and the pace of evolutionary change. While Darwin had struggled to explain the origin of variation and had resorted to mechanisms like pangenesis and the inheritance of acquired characteristics (Darwin, 1868), the Mendelians had a compelling model for the transmission of traits-but lacked a mechanism for adaptive change and natural selection in real populations. This mutual incompleteness delayed synthesis and reinforced antagonism between schools of thought.

As we saw, Darwin himself had acknowledged Lamarckian mechanisms in his later writings. In *The Variation of Animals and Plants Under Domestication* (1868) and *The Expression of the Emotions in Man and Animals* (1872), he speculated that behavioral changes and environmental influences could affect inheritance through use and disuse. These ideas, while reminiscent of Lamarck, were primarily born from the lack of an alternative theory of heredity at the time. They reveal the transitional state of evolutionary theory in the absence of a gene-based understanding of inheritance (Gissis, 2009).

In summary, the early 20th century witnessed a genuine crisis in evolutionary theory: Darwinism lacked a theory of heredity, while Mendelism lacked a mechanism for adaptation. The intense debates between these camps reflected not only empirical disagreements but also divergent methodological and philosophical orientations (Table 2). It would take further theoretical and mathematical developments-particularly in the work of population geneticists-to reconcile these perspectives, but at this juncture, the two schools largely operated in intellectual opposition.

**Table 2-:** Comparative summary of early 20th-century quarrel between Mendelism and Darwinism.

Aspect	Mendelism (Early 20th Century)	Darwinism (Early 20th Century)
View of Variation	Discontinuous variation; traits inherited as discrete units (genes)	Continuous variation; small, incremental changes
Mechanism of Inheritance	Clear, gene-based segregation and dominance (Mendel’s laws)	Lacked a precise mechanism; often relied on blended inheritance or pangenesis
Mechanism of Evolutionary Change	Emphasis on mutations (sometimes large/sudden, as in de Vries’ saltationism)	Gradual accumulation of small variations through natural selection
Strengths	Provided a robust model for inheritance; explained how traits are transmitted	Provided a powerful mechanism for adaptation and the shaping of populations over time
Weaknesses	Lacked an explanation for adaptation in natural populations	Lacked a workable theory of heredity
Philosophical Orientation	Mechanistic, gene-centered, often experimental	Naturalistic, population-level, often statistical and observational
Main Proponents	William Bateson, Hugo de Vries, Carl Correns, Erich von Tschermak	W. F. R. Weldon, Karl Pearson, traditional Darwinists
Criticism of the Other View	Saw Darwinism as vague, outdated, and lacking genetic basis	Viewed Mendelism as too simplistic, based on limited lab traits, and disconnected from natural populations

As recent historiographical work has emphasized, this reconciliation was far from seamless. Gregory Radick (2017, 2023) has shown that the so-called “unification” of Mendelian and Darwinian traditions was not inevitable but rather the product of selective historical pathways that favored particular experimental systems, pedagogical practices, and institutional priorities. Similarly, Charles Pence (2019) has argued that the formalization of population genetics privileged mathematical elegance over biological realism, contributing to a disciplinary culture that equated evolution with statistical changes in allele frequencies. These historiographical insights remind us that the Modern Synthesis, while a triumph of conceptual integration, also codified reductionist assumptions that later movements-such as evo-devo and the Extended Evolutionary Synthesis-have sought to transcend.

### Population genetics and the modern synthesis

The Modern Synthesis (1930s-1950s) represents another of the most significant conceptual shifts in the history of evolutionary biology and achieved the reconciliation between Darwinian natural selection and the Mendelian principles of inheritance, effectively resolving the early 20th-century rift. Through a combination of mathematical theory and empirical research, this synthesis established a unified framework for understanding evolution at the population level.

The foundation of the Modern Synthesis was laid by a group of theoretical population geneticists-Ronald A. Fisher, J.B.S. Haldane, and Sewall Wright-whose work demonstrated that Mendelian inheritance could produce the continuous variation required by Darwinian natural selection. Fisher’s *The Genetical Theory of Natural Selection* (1930) showed that small, heritable mutations, when subjected to selection, could lead to gradual evolutionary change. Haldane (1932) extended these insights by modeling rates of selection and mutation, while Wright (1932) introduced the concept of genetic drift and adaptive landscapes, emphasizing the stochastic nature of evolution in small populations.

The integration of these theoretical advances into a broader biological framework was assisted by Theodosius Dobzhansky’s seminal book Genetics and the Origin of Species (1937), which connected population genetics with natural populations. Dobzhansky and other prominent figures such as Morgan (Morgan, 1910) provided empirical evidence-especially from studies in *Drosophila*-that natural populations contain considerable genetic variation, allowing evolutionary theory to be grounded in real-world data. His formulation established that evolution could be understood as changes in allele frequencies within populations over time.

Julian Huxley played a key role in consolidating these developments through his influential synthesis in *Evolution: The Modern Synthesis* (1942). It was Huxley who coined the term “Modern Synthesis,” framing this integration as a definitive unification of knowledge from genetics, systematics, paleontology, and developmental biology. Huxley’s work emphasized the universality of evolutionary processes and provided a new narrative structure for the life sciences.

Additional contributions were made by Ernst Mayr, whose *Systematics and the Origin of Species* (1942) highlighted the role of reproductive isolation in speciation and defended the biological species concept. George Gaylord Simpson’s Tempo and Mode in Evolution (1944) brought paleontological data into alignment with population-level processes, while G. Ledyard Stebbins extended the synthesis to include plant evolution (Stebbins, 1950).

Together, these efforts established a coherent set of theoretical principles, often summarized as follows: (1) genetic variation arises through random mutation and recombination; (2) evolution consists largely of changes in gene frequencies driven by natural selection; (3) genetic drift plays a role, particularly in small populations; and (4) speciation is the result of the accumulation of genetic differences leading to reproductive isolation (Mayr, 1963). This framework marked a definitive shift from earlier typological and essentialist views of species to a population-based and statistical understanding of evolutionary change.

The Modern Synthesis thus transformed evolutionary biology into a predictive, quantitative science and fostered a widespread consensus across subfields, solving many weaknesses of the original version of Darwinism (Table 3). It unified previously disparate disciplines under a shared theoretical language and methodological approach. As such, it stands as a paradigmatic example of scientific consolidation, with enduring influence across the biological sciences.

**Table 3 -:** Key conceptual issues of early Darwinism and their resolution through the Modern Synthesis.

Difficulties of Early Darwinism	Resolution by the Modern Synthesis
Lack of a hereditary mechanism	Integration of Mendelian genetics showed that discrete genes and alleles could explain inheritance and provide material for natural selection (Fisher, Haldane, Wright).
Difficulty explaining continuous variation	Population genetics demonstrated that Mendelian inheritance, combined with multiple loci, could produce continuous variation required for gradual evolution.
Unclear mechanism for adaptation	Quantitative models and empirical data linked allele frequency changes to natural selection, making adaptation mathematically and empirically tractable.
Limited understanding of speciation	Ernst Mayr emphasized reproductive isolation and the biological species concept, explaining how new species arise through accumulation of genetic differences.
Insufficient integration with paleontology	Simpson and others connected fossil records with population-level processes, showing evolutionary patterns over long timescales.
Focus on animals, neglecting plants	G. Ledyard Stebbins extended evolutionary principles to plant biology, integrating botany into the unified framework.
Lack of predictive and quantitative framework	The Modern Synthesis established a coherent, mathematical, and population-based approach, making evolutionary biology predictive and testable.

### Phylogenetic systematics and the tree of life

The rise of phylogenetic systematics represents one of the most important conceptual developments that consolidated after the Modern Synthesis. Within this updated framework, the need for a systematic, evolutionary-based method for classifying life became increasingly apparent. It was under this intellectual climate that Willi Hennig introduced phylogenetic systematics-commonly known as cladistics-marking a paradigmatic step forward in the logic of biological classification.

Hennig’s work *Grundzüge einer Theorie der phylogenetischen Systematik* was originally published in 1950 and translated to English in 1966 (*Phylogenetic Systematics*, 1966). It provided a methodological and algorithmic foundation for organizing biodiversity based on evolutionary descent. Rather than relying on overall similarity or typological groupings, Hennig proposed that taxa should be classified according to shared derived characters (*synapomorphies*), thus forming monophyletic groups or clades. This reflected the Modern Synthesis’s emphasis on historical and genetic continuity and solved some of its problems (Table 4), aligning taxonomy with the evolutionary processes that the new paradigm described (Hennig, 1966).

**Table 4 -:** Key advances of phylogenetic systematics within the framework of the Modern Synthesis.

Aspect / Feature	Modern Synthesis Contribution	Advancement by Phylogenetic Systematics (Cladistics)
Basis of Classification	Evolutionary change, population genetics, natural selection	Classification based on shared derived characters (synapomorphies), forming monophyletic clades
Conceptual Approach	Descriptive and population-focused, emphasizing gene frequency changes	Hypothesis-driven, logical, testable, reproducible framework for inferring ancestry
Handling of Diversity	Recognizes variation within and between populations	Organizes biodiversity into a hierarchical tree of life reflecting actual evolutionary relationships
Role of Morphology	Morphological traits considered alongside population data	Morphology used selectively; focus on phylogenetic signal rather than overall similarity
Integration with Genetics	Mendelian inheritance and genetic variation underpin evolutionary processes	Molecular markers (DNA/protein sequences) allow precise reconstruction of evolutionary history
Empirical Testing	Population-level patterns observed in natural and lab populations	Parsimony, statistical and computational methods test evolutionary hypotheses at multiple scales
Applications Across Biology	Population genetics, speciation, adaptation	Comparative genomics, evo-devo, conservation biology, ecology, phylogeography
Conceptual Clarity	Provided a unified framework for evolution and adaptation	Operationalizes and extends evolutionary principles into systematic and predictive taxonomy

Cladistics represented a shift from descriptive to hypothesis-driven science, introducing principles such as parsimony, reproducibility, and testability to systematics. It replaced intuition and morphological gradation with a logic-based framework for inferring ancestry, consistent with the population genetics and evolutionary dynamics formalized by the Modern Synthesis (Hull, 1988). The concept of the “tree of life,” once metaphorical, gained mathematical and empirical rigor through this systematic approach.

The development of computational tools such as PAUP (Swofford, 2002), PHYLIP, and later MEGA (Kumar et al., 2018) allowed these principles to be applied to increasingly large and complex datasets. With the emergence of molecular phylogenetics, systematics entered a new era. DNA and protein sequences became standard markers for reconstructing evolutionary history, allowing researchers to test evolutionary hypotheses with unprecedented resolution (Woese et al., 1990).

The alignment between the principles of the Modern Synthesis and the methods of phylogenetic systematics is evident: both stress common descent, inheritance, variation, and the mechanisms of divergence over time. As such, phylogenetic thinking not only operationalized the theoretical commitments of the synthesis but extended them into diverse biological fields. Phylogenies became essential tools in comparative genomics, conservation biology, evolutionary developmental biology (evo-devo), and ecology, reflecting the tree-like structure of life shaped by evolutionary processes.

## Criticisms of the modern synthesis and the rise of the extended evolutionary framework

Despite its enduring contributions, the Modern Synthesis has increasingly been subject to conceptual and empirical criticisms that have stimulated the development of a more inclusive evolutionary framework, commonly referred to as the Extended Evolutionary Synthesis (EES). These challenges do not reject the central tenets of Darwinian evolution or Mendelian inheritance but propose that additional mechanisms-previously neglected or marginalized-must be considered to fully explain biological diversity, development, and adaptation.

A pivotal critique emerged from the work of Motoo Kimura, who in 1968 proposed the neutral theory of molecular evolution. According to Kimura, most nucleotide substitutions at the molecular level are selectively neutral and fix in populations through genetic drift rather than natural selection. This theory redirected evolutionary attention from strictly adaptive processes to stochastic mechanisms, positioning genetic drift as a dominant evolutionary force at the molecular scale (Kimura, 1983). The ensuing debate between neutralists and selectionists became one of the central theoretical controversies in evolutionary biology. Although contemporary views generally accept that both forces act concurrently, there is growing evidence that purifying or negative selection-responsible for removing deleterious mutations-is more prevalent than previously assumed, and may exert a stronger influence on genome evolution than positive selection does (Eyre-Walker and Keightley, 2007; Kern and Hahn, 2018). This challenges the assumption that adaptive natural selection is the primary driver of evolutionary change and highlights the need for a more nuanced view of the evolutionary process.

Simultaneously, the Modern Synthesis has been increasingly critiqued for its genetic reductionism-an approach that posits genes as the sole or principal unit of selection, often treating organisms merely as vehicles for gene propagation. This view, made popular by Richard Dawkins in The Selfish Gene (1976), has been criticized for minimizing the role of developmental, ecological, and cooperative processes in evolution. Critics have pointed out that evolutionary biology must account not only for competition but also for cooperation and symbiosis, which are pervasive in nature (Sender et al., 2016). Studies in endosymbiosis, mutualism, and holobiont theory suggest that many evolutionary transitions, including the origin of eukaryotic cells, are fundamentally cooperative in nature (Margulis, 1970; Margulis and Fester, 1991; Bordenstein and Theis, 2015; Lanier et al., 2017; Vitas and Dobovišek, 2018; Prosdocimi et al., 2019, 2021; Prosdocimi and de Farias, 2024). 

For example, it has been hypothesized that the origin of eukaryotes may trace back to predatory bacteria, such as *Bdellovibrio*, capable of invading larger prokaryotic cells, thereby establishing a stable endosymbiotic relationship that represented a pivotal step in the evolution of eukaryotic cellular complexity (Davidov and Jurkevitch, 2009). For symbiotic organisms to influence host evolution, reliable transmission across generations is essential. In the case of endosymbionts, vertical transmission typically occurs with high fidelity, as these microorganisms are often incorporated directly into the host’s reproductive cells. Notable examples include *Buchnera* spp. in aphid oocytes (Wilkinson et al., 2003), and *Wolbachia* spp. in fruit fly eggs (Ainsworth, 2005). In contrast, exosymbionts are transmitted from parent to offspring via diverse mechanisms, which vary in precision and consistency. In mammals, for instance, symbiotic bacteria are transferred during birth particularly through passage in the vaginal canal (Biasucci et al., 2008) and reinforced through sustained close contact with parents, siblings, and community members (Cooney et al., 2002; Zivkovic et al., 2011). In humans, microbial composition has been shown to be more similar between twin pairs than between twins and their mother, highlighting the role of early shared environments and interactions (Turnbaugh et al., 2009; Reyes et al., 2010). Such microbiota similarities within families or social groups may reflect both genetic relatedness and the influence of early-life microbial exposures. This symbiotic synergy can confer increased fitness to host organisms harboring such symbionts, by enhancing immune defenses or improving the efficiency of nutrient assimilation from available food sources (Dehority, 2003; O’Hara and Shanahan, 2006; Underhill and Iliev, 2014; Grandi and Tramontano, 2018). Moreover, microbial partners can provide hosts with adaptive traits that enhance fitness under specific local environmental conditions. By associating with microbes that are locally adapted, hosts may be able to navigate the fitness landscape more effectively, adjusting their phenotypic expression in response to ecological stressors. This microbial-mediated adaptability can significantly enhance host survival and reproductive success when facing novel or changing environmental pressures. In fact, a growing body of compelling research highlights the significant influence of locally adapted microbes on host phenotypes and overall fitness. A striking example involves bean bugs, which acquire resistance to pesticides by harboring *Burkholderia*-a bacterium capable of degrading these compounds-that naturally occurs in the surrounding soil environment (Kikuchi et al., 2011). Numerous other host organisms similarly exploit their microbiomes to neutralize toxic substances. In ecosystems dominated by creosote plants, for instance, woodrats possess gut microbial communities specialized in breaking down phenolic toxins. Exposure to creosote resin consistently shapes the composition of their microbiome, selectively enriching Actinobacteria with phenol-degrading capabilities, thereby enabling the woodrat to thrive on a chemically defended and otherwise challenging dietary resource (Kohl and Dearing, 2016). 

Another criticism comes from the genomics and systems biology, now mature sciences. It became evident that mapping genotype to phenotype is far more complex than previously understood. Phenotypic traits often result from the interplay of multiple genes (pleiotropy), non-additive gene interactions (epistasis), environmental modulation, and developmental plasticity. These complexities challenge the simplistic view that evolution can be understood solely in terms of allele frequency changes in populations and call for models that incorporate higher-order interactions and emergent properties.

A third major development that has fueled the emergence of the EES is the rise of epigenetics. First introduced by Conrad Waddington in the 1940s and later developed through molecular research, epigenetics refers to heritable changes in gene expression that do not involve alterations in the DNA sequence (Waddington, 1953, 1957). Mechanisms such as DNA methylation, histone modification, and non-coding RNA regulation allow organisms to respond to environmental cues in dynamic ways, and in some cases, these modifications can be passed to offspring (Jablonka and Lamb, 2005; Dolinoy et al., 2007). While the full evolutionary implications of epigenetic inheritance are still being debated, the field has already begun to reshape our understanding of heredity, plasticity, and adaptation - as we shall see.

While these developments have prompted calls for an expanded theoretical framework, it is important to emphasize that the genomic revolution has also provided some of the strongest empirical validations of Darwinian predictions. With the advent of DNA sequencing technologies, evolutionary biologists can now compare entire genomes across species, revealing deep homologies and genetic continuities that strongly support the shared ancestry of life. Molecular phylogenetics has become the gold standard for reconstructing evolutionary histories, and genomic data have corroborated and refined phylogenetic trees initially based on morphological characters (Wiley and Lieberman, 2011). Tools such as whole-genome alignments, molecular clocks, and comparative genomics have allowed scientists to identify conserved genetic elements, patterns of divergence, and signatures of selection across taxa that makes perfect sense and help us to understand the details about the evolutionary processes in many species. These findings demonstrate that the sequences of DNA and proteins not only reflect but also preserve the historical patterns of descent predicted by Darwinian theory. Thus, even as the field evolves to incorporate new insights, genomics continues to reinforce the fundamental principles of evolutionary biology and solidifies the phylogenetic perspective as a cornerstone of modern science.

### Epigenetics: An overlooked pillar of evolution

Among the most profound challenges to the Modern Synthesis, epigenetics has emerged as a central-but often underappreciated-pillar of conceptual transformation in evolutionary biology. While widely recognized in developmental and biomedical research, its evolutionary implications remain relatively marginalized in mainstream discourse. Yet, epigenetics fundamentally alters our understanding of heredity, plasticity, and adaptation, providing a molecular framework for non-genetic inheritance and rapid phenotypic change. 

Epigenetics refers to heritable changes in gene expression that do not involve alterations in the DNA sequence itself. These changes are mediated by biochemical mechanisms such as DNA methylation, histone modifications, and the action of non-coding RNAs, all of which regulate chromatin structure and transcriptional activity (Bird, 2007; Berger et al., 2009). These mechanisms are responsive to environmental stimuli and capable of generating heritable phenotypic variation without any change to the underlying nucleotide sequence.

Among these mechanisms, DNA methylation is the most extensively studied. Methyl groups are typically added to cytosine bases in CpG dinucleotides by DNA methyltransferases (DNMTs), silencing gene expression by blocking transcription factor access or by recruiting repressive protein complexes (Feinberg and Irizarry, 2010). Histone modifications such as acetylation, methylation, and phosphorylation-mediated by enzymes like HATs, HDACs, and HMTs-further modulate the chromatin landscape by altering nucleosome configuration and thereby influencing gene accessibility (Bannister and Kouzarides, 2011). These modifications function in an interdependent network of signaling known as the epigenetic code, adding a new dimension of complexity to gene regulation.

A particularly dynamic aspect of epigenetic regulation is chromatin remodeling, a process involving ATP-dependent multiprotein complexes such as SWI/SNF, ISWI, CHD, and INO80. These complexes can reposition, eject, or restructure nucleosomes to either expose or occlude regulatory DNA regions (Clapier et al., 2017). Chromatin remodeling is essential not only during development and cell differentiation but also in response to environmental and physiological stressors (Smeenk et al., 2010). For example, plants deploy SWI/SNF remodelers like BRAHMA and SPLAYED to activate stress-responsive genes under drought or cold stress (Han et al., 2012). These epigenetic changes can establish stress memory, enabling faster and stronger responses upon future exposures (Lämke and Bäurle, 2017).

The dynamic and reversible nature of chromatin states has been increasingly investigated with modern high-resolution molecular techniques. Tools like ATAC-seq (Assay for Transposase-Accessible Chromatin), DNase-seq, and FAIRE-seq (Formaldehyde-Assisted Isolation of Regulatory Elements) map accessible chromatin regions and identify active regulatory elements with remarkable sensitivity (Boyle et al., 2008; Giresi et al., 2007; Zhang and Pugh, 2011; Buenrostro et al., 2013). MNase-seq allows precise determination of nucleosome positioning, while ChIP-seq (Chromatin Immunoprecipitation sequencing) enables the genome-wide mapping of specific histone modifications and DNA-protein interactions (Barski et al., 2007). Furthermore, techniques such as Hi-C and 3C-derived methods have revolutionized our ability to explore the three-dimensional organization of chromatin, elucidating topologically associating domains (TADs), enhancer-promoter interactions, and large-scale nuclear architecture (Lieberman-Aiden et al., 2009).

These new techniques and findings challenge the gene-centric model of the Modern Synthesis by demonstrating that phenotypic traits and evolutionary adaptations may arise from complex regulatory mechanisms beyond DNA sequence variation. For instance, identical genomes can give rise to dramatically different phenotypes depending on the epigenetic context. This is actually the case of queen and worker bees, whose castes are determined by diet-induced methylation patterns despite genetic identity (Kucharski et al., 2008). Similarly, epigenetic inheritance of epigenetic markers cause transgenerational effects of nutrition, toxins, or stress responses and introduces the idea of a “soft inheritance” that parallels, and at times complements, classical Mendelian transmission (Jablonka and Lamb, 2005; Bonduriansky and Day, 2009).

Thus, epigenetics and chromatin remodeling provide a dynamic and environmentally responsive layer of biological regulation, one that is heritable, reversible, and adaptive. The existence of those mechanisms reshaped our understanding of how traits emerge, persist, and evolve. They emphasize regulatory networks and systems-level interactions rather than linear causality from gene to trait, thus opening new conceptual frontiers in evolutionary theory. 

### Challenges of the post-genomic era to evolutionary theory

Thus, the advent of post-genomic technologies has revolutionized biology, offering unprecedented access to entire genomes and epigenomes across individuals and species. However, this flood of data has also exposed profound challenges to traditional evolutionary frameworks, particularly those rooted in the Modern Synthesis. As evolutionary theory evolves to incorporate these findings, it becomes evident that several foundational assumptions about heredity, mutation, and phenotype require critical re-evaluation.

One of the core challenges in the post-genomic landscape is the elusive definition of the “allele.” Traditionally understood as discrete variants of a gene that influence traits in predictable Mendelian patterns, alleles are increasingly difficult to define unambiguously in the genomic era. Large-scale sequencing projects have revealed that within any given population, the majority of genetic variants are rare or unique (Venter et al., 2001; 1000 Genomes Project Consortium, 2015), and that most individuals carry a high burden of previously uncharacterized variants. This vast allelic diversity complicates the identification of clear-cut genotype-phenotype relationships. Furthermore, genome-wide association studies (GWAS) often detect statistically significant associations that explain only a small fraction of phenotypic variation, a conundrum known as the “missing heritability” problem (Manolio et al., 2009).

Compounding this issue is the realization that individuals do not possess a single, static genome. Instead, humans and other organisms often exhibit genomic mosaicism-variation in the genome within different cells or tissues of the same individual. Mosaicism may arise from somatic mutations, chromosomal rearrangements, or mobile element activity and has been documented in both normal physiology and disease contexts (Poduri et al., 2013). Together with the idea of holobionts, this issue undermined the assumption that one genome sequence can represent an entire organism, posing major challenges for evolutionary models based on population-level genotypic averages.

Further complicating the picture is the presence of extensive homeologous regions-duplicated genomic segments arising from whole-genome duplication events or segmental duplications. These regions are particularly common in plants but also exist in animal genomes, including humans (Dehal and Boore, 2005). Homeologs often retain partial or redundant function, leading to complex patterns of gene expression regulation and compensation. Their evolutionary dynamics-marked by subfunctionalization, neofunctionalization, or gene loss-challenge the gene-centric view of evolution and underscore the importance of genomic architecture in shaping phenotypic traits.

Thus, the mapping of genotype to phenotype has proven to be far more convoluted than early models suggested. Most traits are polygenic, influenced by a network of interacting loci with small effects that are sensitive to environmental modulation and developmental history (Boyle et al., 2017). This complexity calls into question the reductionist notion that evolutionary change can be adequately described by shifts in allele frequencies alone. Instead, it supports a more systems-oriented approach that incorporates regulatory networks, epigenetic landscapes, and ecological feedbacks.

Another major conceptual shift introduced by post-genomic science is the recognition that genetic information is deeply context-dependent. Fields like pharmacogenomics and nutrigenomics have demonstrated that genetic variants affect individual responses to drugs and dietary components in highly personalized ways. For instance, polymorphisms in the cytochrome P450 gene family significantly alter drug metabolism and efficacy among individuals (Ingelman-Sundberg, 2004). Similarly, variations in genes related to lipid metabolism or glucose transport modulate the physiological response to different diets, supporting the development of personalized nutrition plans (Ordovas and Corella, 2004). These insights reveal that selection pressures may operate in highly individualized contexts, challenging universal models of adaptive fitness.

## Extended synthesis basic principles

Thus, the critiques addressed in the previous sections invite a central question: does the growing body of evidence concerning epigenetic inheritance, plasticity, symbiosis, and ecological feedbacks justify the designation of a new evolutionary paradigm? Over the past two decades, the field of evolutionary biology has undergone significant conceptual expansion, giving rise to what is now referred to as the Extended Evolutionary Synthesis (EES) (Smocovitis, 1996). The EES model seeks to broaden evolutionary theory by incorporating a wider array of mechanisms that influence heritability, variation, and adaptation. These include epigenetic regulation, phenotypic plasticity, niche construction, symbiosis, and behavioral inheritance, among others. Table 5 outlines the core concepts of the EES, highlighting their theoretical significance, key contributors, and biological implications.

**Table 5-:** Core concepts of the Extended Evolutionary Synthesis (EES).

Concept / Mechanism	Description	Biological Implications	Key Authors/Sources
Phenotypic Plasticity	Capacity of a genotype to produce different phenotypes in response to environmental cues	Allows rapid, reversible adaptation; may precede genetic changes	West-Eberhard (2003)
Epigenetic Inheritance	Transmission of gene expression patterns via DNA methylation, histone modification, etc.	Enables non-genetic adaptation; revives soft inheritance concepts	Jablonka and Lamb (2005); Bonduriansky and Day (2009)
Developmental Bias	Certain phenotypes are more likely due to constraints or propensities in developmental systems	Not all variation is random; evolution is shaped by developmental dynamics	Müller (2007); Pigliucci (2007)
Niche Construction	Organisms modify their environments in ways that influence their own evolution	Feedback loop between organisms and environments; breaks nature/nurture dualism	Odling-Smee et al. (2003)
Inclusive Inheritance	Broadens heredity to include genetic, epigenetic, ecological, and cultural transmissions	Inheritance is multilayered and context-dependent	Laland et al. (2015)
Symbiosis and Holobionts	Organisms evolve in tight association with symbiotic partners (microbiome, virome, etc.)	Challenges individualistic views of evolution; emphasizes community-level selection	Gilbert et al. (2012b); Bordenstein and Theis (2015)
Facilitated Variation	Conserved core processes allow evolutionary innovation through recombination of modular systems	Explains evolvability and the emergence of complexity	Gerhart and Kirschner (2007)
Cultural and Behavioral Inheritance	Behaviors and learned information passed across generations influencing fitness	Particularly important in humans and other social species	Laland and Brown (2002)
Reciprocal Causation	Evolutionary processes involve feedbacks where cause and effect are not strictly linear	Evolution is dynamic, co-constructed, and context-sensitive	Laland et al. (2015); Uller and Laland (2019)
Extended Organism	Organisms build structures (nests, burrows, webs) that influence selection and are part of phenotype	Redefines phenotype to include ecological structures	Jablonka and Lamb (2005); Dawkins (2004)
Dissipative Structures	Organisms as open, far-from-equilibrium systems that sustain internal order by continuous energy throughput and entropy export	Embeds thermodynamic constraints in evolutionary innovation; explains emergence of hierarchical complexity via non-equilibrium dynamics	Prigogine and Stengers (1984); Kleidon and Lorenz (2005); Kondepudi et al. (2020)

Align with EES, there is a growing interest in what Philip Ball (2023) called *New Biology,* reflecting a profound re-evaluation of life’s principles in the post-genomic era. Ball argues that biology is undergoing a conceptual transformation comparable in depth to the Modern Synthesis-one that abandons strict genetic determinism and reductionist frameworks in favor of relational, dynamic, and emergent understandings of living systems. The *New Biology* emphasizes the organism as an integrated whole, shaped by networks of interactions that span from molecules to ecosystems. This perspective aligns with the EES by recognizing that inheritance, adaptation, and evolution operate through multiple channels-genetic, epigenetic, ecological, and symbolic (Jablonka and Lamb, 2005). As Ball notes, life’s essence lies not in static blueprints encoded in DNA, but in the self-organizing, context-dependent processes that sustain biological order (Ball, 2023). This view converges with the holodarwinian perspective we propose here, in which the study of evolution becomes inseparable from the study of complexity and interdependence.

### Niche construction, ecological feedbacks and epigenetic inheritance

One of the clearest empirical illustrations of niche construction comes from eusocial insects such as termites and ants, whose colony-building behavior actively shapes the microclimatic and chemical properties of their environment. For example, termite mounds can regulate temperature and humidity through complex ventilation systems, creating stable internal environments that buffer against external climatic fluctuations (Turner, 2001). These structures, while built through behavioral processes, exert selective pressures on both the termites and associated microbial communities, illustrating the feedback loops central to niche construction theory (Odling-Smee et al., 2003).

Among the conceptual pillars of the Extended Evolutionary Synthesis (EES), epigenetics has arguably been the most transformative-and yet remains one of the least incorporated into mainstream evolutionary thinking. As discussed earlier, epigenetic regulation refers to heritable changes in gene expression that do not involve alterations in the DNA sequence itself, but are orchestrated by mechanisms that modulate chromatin conformation and accessibility, including DNA methylation, histone modification, and non-coding RNAs (Bird, 2007; Berger et al., 2009). These processes add a critical regulatory layer to the genome, enabling organisms to respond dynamically to environmental stimuli and developmental cues.

Evidence indicates that epigenetic marks can be stable across cell divisions and, in some instances, transmitted across generations-a phenomenon described as “soft inheritance” (Bonduriansky and Day, 2009). These heritable modifications form a kind of “cellular memory” that parallels Lamarckian ideas of the inheritance of acquired characteristics, albeit via distinct molecular mechanisms. Modern epigenetic inheritance is supported by empirically verified biochemical pathways responsive to environmental changes. Nevertheless, this revival of Lamarckian motifs-often termed *neolamarckism*-remains a topic of debate, particularly concerning the long-term stability and evolutionary relevance of such mechanisms (Richards et al., 2017).

A striking example of transgenerational epigenetic inheritance has been reported in *Arabidopsis thaliana*, where spontaneous epimutations-changes in DNA methylation patterns not linked to nucleotide mutations-were shown to accumulate at a rate significantly higher than genetic mutations. These heritable epigenetic variants influenced gene expression and phenotypic traits such as flowering time and stress response, independently of DNA sequence changes (Becker et al., 2011; Schmitz et al., 2011). Such findings highlight the existence of a parallel system of heritable variation with potential evolutionary relevance, particularly in rapidly changing environments where genetic adaptation may lag.

### Phenotypic plasticity and genetic assimilation

Closely intertwined with epigenetics is the phenomenon of phenotypic plasticity-the capacity of a single genotype to produce a range of phenotypes in response to varying environmental conditions. Once considered a marginal topic, plasticity is now recognized as a central feature of ecological and evolutionary processes (Pigliucci, 2001). The EES places renewed emphasis on its evolutionary significance, arguing that plastic responses can enable facilitated variation capable to produce rapid adaptation even in the absence of genetic change.

Well-known examples include the water flea *Daphnia*, which develops protective spines only when exposed to predator cues (Agrawal et al., 1999), and the butterfly *Bicyclus anynana*, whose wing patterns change depending on the season (Brakefield et al., 2009). In plants, chromatin remodeling complexes such as BRAHMA and SPLAYED are recruited to activate stress-responsive genes, leading to improved resilience in subsequent stress events (Han et al., 2012; Lämke and Bäurle, 2017). In animals, chromatin remodeling plays essential roles in tissue specification, as exemplified by the interaction of SWI/SNF with MyoD in muscle cells and CREST-BRG1 in neuronal differentiation (de la Serna *et al*., 2006; Ho and Crabtree, 2010).

These plastic responses can become genetically assimilated over time whereby initially environmentally induced traits become encoded in the genome through selection, blurring the boundary between plasticity and genetic evolution. Far from being buffers, plastic traits can generate novel phenotypic variation and open new evolutionary pathways, providing raw material for natural selection.

### Behavioral, cultural, and symbolic inheritance

Importantly, the EES extends this understanding of inheritance beyond the molecular and developmental levels to include behavioral and symbolic dimensions, particularly in species with complex cognition and sociality. Behavioral inheritance refers to the transmission of learned behaviors across generations via social learning rather than genetic encoding.

In birds, such as the zebra finch, vocal learning is transmitted from parent to offspring and modulated by social environment, exemplifying behavioral heredity with fitness consequences (Catchpole and Slater, 2008).

In primates, young individuals learn foraging techniques, tool use, and social rules through imitation and observation, forming cultural traditions that persist over time (Whiten et al., 1999). By virtue of the very cognitive evolution shaped by neural regions that have specifically evolved to enhance imitative behaviors and to confer upon them a social and empathic significance, thereby facilitating learning, this process is encapsulated within the framework of *Theory of Mind* (Rizzolatti and Craighero, 2004). Cavalli-Sforza and Feldman proposed that social learning enhances human adaptability by reducing the costs associated with individual learning, particularly those linked to trial-and-error strategies. Since individual learning can be time-consuming and error-prone, acquiring information from others who have already incurred those costs provides a more efficient alternative. Through imitation, individuals can access a substantial body of adaptive knowledge without bearing the energetic or cognitive burdens of independently discovering and validating that information (Cavalli-Sforza and Feldman, 1981). This process, acting in conjunction and synergy with neuroevolutionary mechanisms such as the mirror neuron system, has contributed to greater group resilience, ultimately resulting in enhanced species-level fitness. 

In humans, cultural evolution and symbolic inheritance play an even more pronounced role. Language, beliefs, and symbolic practices are passed from generation to generation, forming cumulative traditions that influence not only individual behavior but also group structure, ecological strategies, and even evolutionary pressures (Corning, 2008, 2022). The co-evolution of genes and culture demonstrates how symbolic systems can become evolutionary forces that shape environments and organize feedback mechanisms that influence selection (Jablonka and Lamb, 2005; Laland et al., 2010).

These forms of behavioral and symbolic transmission named as inclusive inheritance resonate with a broader *neolamarckian* perspective, in which acquired characteristics-be they molecular, developmental, behavioral, or cultural-can influence the evolutionary process across multiple timescales and inheritance systems. While the molecular mechanisms may differ, the core idea that inheritance is not limited to DNA sequence changes (but includes dynamically acquired and transmitted traits) forms a crucial dimension of the EES.

Symbolic and cultural inheritance therefore represent non-genetic systems of transmission, particularly in humans and other cognitively complex species. Behaviors such as tool use in chimpanzees, dialect learning in songbirds, and language acquisition in humans exemplify the continuity of information beyond DNA (Laland and Galef, 2009; Henrich, 2016). Cultural evolution can introduce rapid behavioral adaptation and, in some cases, shape selective environments for future generations-an idea increasingly explored within cultural niche construction theory. However, while these forms of inheritance are recognized as evolutionary relevant, their integration into a unified evolutionary framework remains an open challenge, especially regarding modeling fidelity, generational transmission rates, and interaction with genetic evolution. 

Knowledge transmission constitutes a primary factor underpinning the potential deployment of tools aimed at achieving adaptive or utilitarian benefits (Mann et al., 2008). In humans, cultural pressure is particularly prominent. Evolutionary pressures, coupled with random mutations, have led to the development of an exceptionally complex and efficient nervous system. This has enabled *Homo sapiens* to engage in more sophisticated communication, abstract thinking, and the emergence of technology. In some point, technology has acted as a catalyst for the species’ evolutionary potential. Through a form of positive feedback, the human capacity to generate and share knowledge has allowed technological advancements to be disseminated across increasingly larger groups. This process has driven exponential growth in technological complexity, following a pattern analogous to horizontal gene transfer. Also, the rate of technological development far outpaces that of biological evolution, thereby enhancing human fitness and helping to explain the increasingly rapid rise in *Homo sapiens*’ adaptive success. A reflection on the exponential changes observed over the past two centuries illustrates the profound impact of cultural and technological evolution in humans: (i) human population has undergone a dramatic increase-from approximately 1 billion individuals in the 19th century to over 8 billion today-highlighting a marked rise in reproductive success and survival (Ritchie *et al*., 2023); (ii) in conjunction this demographic expansion has been associated with a decline of mortality and has been largely driven by advances in medicine, public health, and scientific research. Life expectancy has significantly increased over the past century; however, (iii) over the last 200 years, *Homo sapiens* has dramatically expanded its ecological footprint, effectively modifying and occupying nearly all terrestrial environments. In just the past two decades, the anthropogenic mass-comprising all human-made materials-has approximately doubled (Elhacham et al., 2020). Thus, within the framework of the Extended Evolutionary Synthesis (EES), technology may be regarded as a promoter or catalyst of cultural change and knowledge sharing. Could we measure somehow the relative contribution of technological factors to evolutionary dynamics? 

### Eco-Evo-Devo and thermodinamic perspectives

Additionally, a whole field of Eco-Evo-Devo also encourages a systems-level view in which developmental pathways are not only shaped by ecological conditions but can also feedback to restructure ecosystems themselves, creating reciprocal causation between organisms and their environments. This perspective highlights how selection pressures can be modified by developmental plasticity, leading to context-dependent trajectories of evolution (Sultan, 2007). Furthermore, Eco-Evo-Devo integrates insights from comparative embryology, ecological epigenetics, and environmental physiology to understand how conserved genetic toolkits generate diverse morphological outcomes under different environmental regimes (Carroll, 2005). By emphasizing modularity, heterochrony, and developmental bias, the framework reveals how certain phenotypic variants are more readily produced than others-not due to selection alone, but because of inherent constraints and affordances in developmental architecture. As such, Eco-Evo-Devo not only extends evolutionary theory but also deepens our understanding of the origins of innovation and constraint in evolutionary trajectories.

Within the framework of the Extended Evolutionary Synthesis (EES), living organisms are reconceived as open, far-from-equilibrium thermodynamic systems that continuously exchange energy and matter with their environment. To sustain internal order they absorb free energy (e.g. nutrients or solar radiation) and export entropy as heat and metabolic waste, thereby increasing global entropy while maintaining locally low entropy (Prigogine and Stengers, 1984; Schrödinger, 1944). This process of “ordered dissipation” is encapsulated by Prigogine’s dissipative-structure theory, which demonstrates how non-equilibrium systems can spontaneously sustain organized states through continuous energy fluxes (Prigogine and Stengers, 1984). Far from being passive, these fluxes couple organisms to environmental gradients and intertwine with niche-construction dynamics: by actively modifying their habitats (for instance via burrowing, secretion, or soil alteration), organisms reshape the thermal and chemical gradients that govern selective pressures and evolutionary (Odling-Smee et al., 2003).

Prigogine further showed that under far-from-equilibrium conditions, microscopic fluctuations may be amplified by energy flows to produce novel ordered structures-a phenomenon termed “order through fluctuations.” The same principle seen in Bénard convection cells applies to biological populations, where random mutations amplified by selection yield phenotypic innovations (Prigogine and Stengers, 1984; Kondepudi et al., 2020). Accordingly, the major transitions in evolution-from the origin of the eukaryotic cell to the rise of complex social entities-require not only genetic variation but also sufficient dissipative fluxes to support emergent levels of organization (Prigogine and Stengers, 1984; Kondepudi *et al*., 2020).

Finally, some investigators have proposed the Maximum Entropy Production Principle (MEPP) as an evolutionary guide, whereby living systems preferentially adopt configurations that dissipate energy at the highest rate compatible with survival (Kleidon, 2005). Although MEPP remains controversial and context-dependent, it shifts emphasis onto the physico-dissipative facets of living systems, underscoring how biological complexity can emerge from a dynamic trade-off between internal order and external dissipation (Weber et al., 1990). For example, the independent evolution of endothermy in mammals and birds vividly illustrates this principle at the individual level: by shifting from an ectothermic reliance on ambient temperature to metabolically generated internal heat production, these lineages achieved basal energy-dissipation rates far exceeding those of similarly sized ectotherms. This metabolic innovation was positively selected because it enabled precise thermoregulation across diverse climates and daily cycles, thereby unlocking vast new ecological opportunities. From an entropy-production standpoint, endothermy constitutes a configuration that maximizes entropy export-through exceptionally high fluxes of oxygen and nutrients-while maintaining the internal order necessary for survival and reproduction; the resulting interplay between sophisticated thermoregulatory networks and enhanced heat dissipation exemplifies how complex physiological traits can arise from the balance of order and dissipation (Kleidon, 2005; Weber *et al.*, 1990).

Taken together, these insights reconfigure our understanding of evolution as a multidimensional and multilevel process. Eco-evo-devo, epigenetics, phenotypic plasticity, chromatin remodeling, behavioral learning, symbolic inheritance, and the view of organisms as dissipative structures collectively demonstrate that evolutionary change can originate not only from random genetic mutation and classical selection but also from responsive, regulatory, and socially transmitted mechanisms. These findings challenge the reductionist assumptions of the Modern Synthesis and demand a richer, more integrative theoretical framework that fully integrates genetic, epigenetic, ecological, behavioral, and thermodynamic perspectives.

## Criticisms and limitations of the extended evolutionary synthesis

While the Extended Evolutionary Synthesis (EES) has generated enthusiasm for broadening evolutionary theory, it has also faced criticism regarding its conceptual coherence, empirical necessity, and explanatory power. The main lines of criticisms are listed on Table 6. Thus, some prominent evolutionary biologists argue that the mechanisms proposed by the EES-such as epigenetic inheritance, niche construction, and developmental plasticity-do not warrant a theoretical renovation of the Modern Synthesis but can instead be accommodated within its existing framework.

**Table 6-:** Main criticisms and limitations of the Extended Evolutionary Synthesis (EES).

Criticism	Description	Main References
Lack of Novel Predictive Power	EES mechanisms have not yet generated unique or superior predictions compared to those of the Modern Synthesis.	Wray et al. (2014)
Sufficient Flexibility of Modern Synthesis	Existing evolutionary theory is seen as adaptable enough to integrate new findings without a paradigm shift.	Wray et al. (2014); Charlesworth et al. (2017)
Insufficient Quantitative and Population Models	EES lacks rigorous mathematical models equivalent to those in population genetics, especially for epigenetics and soft inheritance.	Lynch (2007); Charlesworth et al. (2017)
Conceptual Ambiguity	Critics argue that EES often conflates proximate and ultimate causes, especially regarding plasticity and developmental bias.	Dickins and Rahman (2012)
Lack of Empirical Necessity	The empirical phenomena highlighted by EES are seen as addressable within the current framework and do not demand theoretical reconstruction.	Charlesworth et al. (2017); Futuyma and Kirkpatrick (2017)
Overstatement of Novelty	Many EES concepts (e.g., cultural transmission, horizontal gene transfer) have long been studied within evolutionary biology.	Griffiths et al. (2008)
Limited Theoretical Integration	Although EES brings attention to new processes, it lacks formal integration into models that allow for predictive and testable outcomes.	Lynch (2007); Charlesworth et al. (2017)

One of the most prominent critiques has been articulated by Wray et al. (2014), who contend that the EES has yet to demonstrate novel predictions or explanatory capacities that surpass those of the Modern Synthesis. According to them, the Modern Synthesis remains sufficiently flexible to integrate emerging findings without the need for a paradigm shift. Similarly, Charlesworth, Barton, and Charlesworth (2017) argue that many EES mechanisms lack the population-genetic rigor and empirical quantification that underpin the Modern Synthesis. They caution that enthusiasm for conceptual expansion must not come at the cost of testability and formalization, both of which are essential for a theory to function scientifically.

Moreover, critics highlight that the EES often blurs the distinction between proximate and ultimate causes, especially when emphasizing plasticity and developmental bias (Dickins and Rahman, 2012). While these factors undoubtedly shape phenotypic outcomes, critics question whether they fundamentally alter evolutionary trajectories in a way that demands theoretical reconstruction. In this view, developmental constraints or ecological feedbacks may influence the distribution of variation on which selection acts, but do not replace the central role of selection in driving evolutionary change.

Another common objection concerns the lack of integrative mathematical models and quantitative tools within the EES framework. Unlike population genetics, which provides well-established models to predict allele frequency dynamics, EES proponents have yet to produce analogous models for phenomena like epigenetic inheritance or symbiotic selection (Lynch, 2007; Futuyma and Kirkpatrick, 2017). This methodological gap has led some to view the EES more as a conceptual program than a predictive scientific theory.

Despite the growing empirical evidence for epigenetic inheritance and developmental plasticity, these phenomena still face important challenges when it comes to formal integration into evolutionary models. As Charlesworth et al. (2017) note, most models of population genetics are still not equipped to quantify how epimutations propagate through populations or contribute to long-term adaptation. Similarly, Lynch (2007) has emphasized that the absence of predictive mathematical frameworks limits the evolutionary relevance of soft inheritance beyond anecdotal or case-specific observations. Therefore, while the Extended Synthesis rightly draws attention to these mechanisms, a full integration requires theoretical tools as rigorous and testable as those of classical genetics.

Additionally, some critics point out that many phenomena highlighted by EES advocates-such as cultural transmission or horizontal gene transfer-are not entirely new and have been studied under the umbrella of evolutionary theory for decades (Griffiths et al., 2008). From this perspective, the EES may risk overstating its novelty by repackaging existing ideas rather than offering transformative insights.

Thus, while the EES has brought attention to important and often neglected processes in evolution, it remains contested whether these processes justify the label of a new synthesis. Critics call for clearer definitions, predictive models, and empirical demonstrations that EES mechanisms significantly reshape evolutionary outcomes. Until then, the EES may remain, in the eyes of its skeptics, a valuable but supplementary perspective rather than a full-fledged paradigm shift.

## Discussion

Thus, the historical development of theories in evolutionary theory have revealed a dynamic and pluralistic intellectual landscape rather than a linear progression of unified concepts (Table 7). As this article has shown, Darwinian natural selection, the rediscovery of Mendelian genetics, and the integration of population genetics in the Modern Synthesis each marked critical milestones in establishing a scientifically coherent view of biological change. However, the enduring strength of evolutionary theory lies not in its stability, but in its capacity to adapt to new empirical insights and conceptual frameworks. The contemporary memeplex of Darwinism as Modern Synthesis has been inundated by the flood of data coming from many different intellectual crossroads in multiple biological disciplines, providing a more holistic view of evolutionary biology.

**Table 7-:** Comparation of the main paradigms of evolutionary biology, highlighting the central theoretical changes that occurred between the four main milestones.

Paradigm \ Concepts	Natural Theology	Classical Darwinism	Modern Synthesis	Extended Evolutionary Synthesis
Approximate Period	400-1859	1859-early 20th century	1930s-1970s	2000s-present
View of Nature	Pre-established harmony; divine creation	Nature as competitive and shaped by natural selection	Nature governed by genes and selective pressures	Nature as dynamic, multilevel, and co-constructed
Unit of Evolution	Fixed species	Populations	Genes	Organisms + environments + symbioses (holobionts)
Main Mechanism of Change	Divine plan / adaptation as proof of God	Natural selection acting on individual variation	Mutation + recombination + natural selection	Selection + plasticity + epigenetics + niche construction + symbiosis
Origin of Variation	Not applicable	Random variation (not explained)	Random genetic mutations	Genetic, epigenetic, behavioral, and ecological variation
Heredity	Essentialist / fixist	Not fully understood	Mendelian inheritance / population genetics	Genetic + epigenetic + ecological and symbiotic transmission
Evolutionary Time	Static	Gradual and continuous	Slow and incremental	Can be rapid, discontinuous, with reversals or pulses
Level of Selection	Not applicable	Individuals	Genes / individuals / populations	Multiple levels: genes, individuals, groups, ecosystems
Adaptation	Intentional design	Result of natural selection	Functional optimization through selection	May also arise through plasticity, symbiosis, exaptation
Organism-Environment Relationship	Passive and fixed environment	Environment selects the fittest	Environmental pressure on the organism	Organisms and environments co-construct each other
Role of Development	Not recognized	Ignored	Ignored (genes seen a independent of development)	Central: evo-devo, epigenetics, phenotypic plasticity
Importance of Symbiosis	Unknown	Ignored	Marginal	Crucial: holobionts, horizontal gene transfer, virome
Thermodynamic Dimension	Not applicable	Not addressed	Not addressed	Organisms as open, far-from-equilibrium systems (dissipative structures); continuous energy flux and entropy export
Key Thinkers	William Paley	Charles Darwin, Alfred Wallace	Dobzhansky, Mayr, Simpson, Haldane	Laland, Jablonka, West-Eberhard, Gilbert, Noble, Odling-Smee

The emergence of the Extended Evolutionary Synthesis (EES) is best understood not as a paradigmatic rupture, but as a reflective shift in emphasis-a conceptual realignment that brings previously peripheral mechanisms into the center of evolutionary discourse. In this sense, the EES reframes the field: it does not discard natural selection or Mendelian inheritance, but contextualizes them within broader systems of developmental, ecological, and symbiotic interaction (Müller, 2007; Laland et al., 2015).

One of the most significant contributions of the EES is its insistence on multilevel and multidimensional inheritance. The rise of epigenetics and phenotypic plasticity has complicated the gene-centric model inherited from the Modern Synthesis, showing that environmental inputs can produce heritable changes in phenotype without altering the underlying DNA sequence (Jablonka and Lamb, 2005; Bonduriansky and Day, 2009). This “soft inheritance” does not undermine Mendelian principles, but reveals parallel layers of transmission that interact with genetic information in context-dependent ways. Additionally, organisms can be viewed as dissipative structures-open, far-from-equilibrium systems that maintain internal order by importing free energy and exporting entropy-so that energy fluxes themselves channel and facilitate evolutionary innovation (Prigogine and Stengers, 1984; Kondepudi et al., 2020). This thermodynamic dimension further underscores the need for an integrative framework that unites genetic, developmental, ecological, and energetic perspectives

Moreover, the EES foregrounds organism-environment reciprocity through concepts such as niche construction, in which organisms actively modify their ecological contexts, feeding back into the selective pressures they experience (Odling-Smee et al., 2003). Similarly, the holobiont concept dissolves the boundaries of the individual, proposing that evolution often acts on assemblages of hosts and symbiotic microbes, with collective phenotypes and co-evolving genomic networks (Rosenberg and Zilber-Rosenberg, 2008; Gilbert et al., 2012b).

These ideas collectively encourage a systems-oriented view of evolution. Rather than viewing evolutionary change as the accumulation of small genetic mutations shaped by external selection, the EES invites models in which developmental bias, regulatory networks, horizontal gene transfer, cultural transmission, and eco-evo-devo dynamics play central roles. This broader framework reopens space for a pluralistic understanding of causality and inheritance in evolutionary processes.

However, the EES is not without its limitations. As critics have noted, many of its core concepts lack the formal mathematical rigor of population genetics (Wray et al., 2014; Charlesworth et al., 2017), and few have produced predictive models that rival those of the Modern Synthesis. Moreover, the epistemological challenge of integrating systemic, emergent, and context-sensitive mechanisms into testable frameworks remains substantial (Lynch, 2007; Dickins and Rahman, 2012). In this respect, the EES may still be more of a programmatic agenda than a settled theory.

Despite these challenges, the EES offers an intellectually generative lens that better aligns with the complexity of contemporary biology. The increasing emphasis on inclusive inheritance-encompassing genetic, epigenetic, ecological, behavioral, and symbolic domains-calls for a richer explanatory architecture in the life sciences (Griffiths et al., 2008; Uller and Laland, 2019). These developments, grounded in empirical advances, suggest that evolution is not merely a competition of replicators, but a dynamic negotiation between organisms, symbionts and their environments across multiple temporal and spatial scales.

In conclusion, the Extended Evolutionary Synthesis should be viewed as a natural outgrowth of the post-genomic and post-reductionist era of biology. It honors the Darwinian tradition while expanding its explanatory scope. Whether or not it becomes a fully formalized synthesis akin to the Modern Synthesis, its contributions to theory and practice are already reshaping the questions we ask, the mechanisms we explore, and the boundaries we draw in evolutionary science.

## Final reflections: Evolutionary biology as an intellectual heritage

**Figure 1-:**
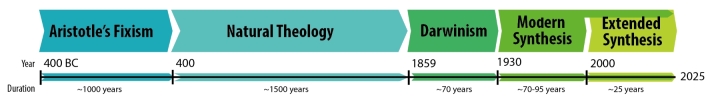
Intellectual lineage of evolutionary theories over the past 2,500 years. Box sizes are not to scale. The Modern Synthesis is considered to coexist with the Extended Synthesis in contemporary biology.

From its theological and philosophical roots to its genomic and ecological expansions, evolutionary biology stands as one of the most profound achievements of the human intellect (Figure 1). The journey from natural theology-where purpose and design were attributed to a divine will modeled on human intention-to a modern scientific framework grounded in variation, heredity, and natural processes marks a relevant shift in humanity’s understanding of its place in nature.

Given the remarkable longevity of Natural Theology as the dominant explanatory framework in Western thought-persisting in various forms from Aristotle to Darwin-it is perhaps unsurprising that certain groups continue to defend it, more as an expression of cultural and intellectual inertia than as a viable scientific alternative. Yet, its underlying premises remain fundamentally at odds with the materialistic and mechanistic foundations of contemporary science. By invoking an undefined and capricious supernatural designer to account for complexity, Intelligent Design reintroduces the explanatory circularity that science has long worked to move beyond: complexity exists because it was designed, and it was designed because it is complex. In doing so, it offers little in the way of genuine explanatory power, ultimately functioning as a philosophical or theological statement often dressed in scientific language. While the teleological reasoning of Natural Theology has long been replaced by mechanistic explanations, Paley’s watchmaker argument continues to haunt modern science. The impressive complexity and interdependence of biological systems often transcend the explanatory power of current biochemical and genetic models, suggesting that the mystery of organization in living beings remains only partially unveiled. This acknowledgment-that science can only illuminate part of nature’s vast phenomena while leaving others insufficiently understood-also underscores the enduring difficulty of comprehending life in its full complexity. Modern biology, for all its analytical power, often fragments the living world into isolated mechanisms, overlooking the integrative and emergent properties that characterize living systems. Such reductionist perspectives have been essential for experimental progress, yet they also risk obscuring the complex and dynamic nature of biological organization. In this sense, the dialogue between the measurable and the ineffable remains open.

Darwin’s On the Origin of Species (1859) inaugurated not only a new science but a new worldview, one that decentered the human and redefined life as an interconnected, dynamic, and contingent process. The Modern Synthesis further solidified this vision by integrating Mendelian genetics, population biology, and selection theory into a coherent explanatory structure. The development of cladistic methods and later molecular phylogenetics placed systematics on an explicit, testable footing, enabling robust evolutionary inference through hypothesis-driven frameworks (Hennig, 1966; Swofford, 2002; Kumar et al., 2018). Those methods provided a *bona fide* basis for the study of biogeography, and allowed great insights into the understanding of populations, clades and ecosystems evolution. 

Today, the rise of the Extended Evolutionary Synthesis continues this legacy by acknowledging the richness of inheritance systems, the plasticity of development, and the reciprocal agency of organisms within their environments. Recent scholarship has explicitly addressed both progress and continuing controversies about the scope and status of the Extended Evolutionary Synthesis. Philosophers and historians have clarified what is at stake in the debate and urged pragmatic criteria for success (Lewens, 2019). Empirical syntheses and community reports produced by the EES initiative document continuing advances across plasticity, niche construction, non-genetic inheritance, and eco-evo-devo while acknowledging the need for more formal models (EES, 2021). Complementary analyses emphasize theoretical plurality within evolutionary biology and the practical benefits of integrating developmental and systems perspectives (Prentiss, 2021). At the same time, popular and synthetic treatments of the “New Biology” argue that post-genomic approaches are reshaping foundational concepts and habits of explanation in the life sciences (Ball, 2023). Taken together, these recent contributions make two points clear: (i) the EES has matured into a substantive, empirically grounded research program whose claims are now being tested across diverse taxa, and (ii) the main methodological challenge identified by critics-the development of explicit, predictive formal models-remains an active frontier for current research (Laland et al., 2015; Lewens, 2019; Prentiss, 2021; EES, 2021; Ball, 2023).

As this article has explored, the history of evolutionary thought is not merely a sequence of scientific advances-it is also a record of changing metaphors, epistemologies, and conceptual frameworks. It reflects a continual dialogue between philosophical reflections, shaped by innovation and cultural context. In this sense, evolutionary biology is more than a scientific discipline: it is a shared intellectual heritage, illuminating the dynamic processes that link all living beings across time. Its historical trajectory teaches us that science progresses not by eliminating its past, but by revisiting, revising, and expanding it in the light of new knowledge. The Extended Evolutionary Synthesis, far from erasing Darwin or the Modern Synthesis, participates in this tradition of cumulative enrichment. Recognizing this history as part of the cultural and intellectual patrimony of humanity not only deepens our appreciation for the science of evolution, but also reminds us that our current models-however powerful-are provisional. They are steppingstones toward a more comprehensive, pluralistic, and ecologically grounded understanding of the complex, marvelous and intriguing living systems.

## Data Availability

 This study is theoretical in nature and did not generate or analyze new datasets.
